# Cannabis use motives and associations with personal and work characteristics among Canadian workers: a cross-sectional study

**DOI:** 10.1186/s12995-024-00424-7

**Published:** 2024-06-13

**Authors:** Nancy Carnide, Bethany R. Chrystoja, Hyunmi Lee, Andrea D. Furlan, Peter M. Smith

**Affiliations:** 1 Institute for Work & Health, 400 University Avenue, Suite 1800, Toronto, ON M5G 1S5 Canada; 2 Dalla Lana School of Public Health, University of Toronto, 155 College Street, 6th floor, Toronto, ON M5T 3M7 Canada; 3 Cumming School of Medicine, University of Calgary, 3330 Hospital Drive NW, Calgary, AB T2N 4N1 Canada; 4 Toronto Rehabilitation Institute, University Health Network, 550 University Avenue, room 7- 141, Toronto, ON M5G 2A2 Canada; 5 Department of Medicine, Temerty Faculty of Medicine, University of Toronto, C. David Naylor Building, 6 Queen’s Park Crescent West, 3rd floor, Toronto, ON M5S 3H2 Canada; 6 Department of Epidemiology and Preventive Medicine, Monash University, 553 St Kilda Road, Melbourne, VIC 3004 Australia

**Keywords:** Cannabis, Motives, Work-related, Workers, Work, Reasons, Marijuana, Workplace, Cross-sectional, Canada

## Abstract

**Background:**

Research on cannabis use motives has focused on youth. Little is known about motives among working adults, including how work may play a role. This study aimed to describe cannabis use motives and their connection to work, and identify the personal and work correlates of work-related motives among a sample of workers.

**Methods:**

A national, cross-sectional sample of Canadian workers were queried about their cannabis use. Workers reporting past-year cannabis use (*n* = 589) were asked their motives for using cannabis and whether each motive was related to work or helped them manage at work (i.e., work-related). Multinomial logistic regression analyses were conducted to estimate the associations of personal and work characteristics with work-related cannabis use motives (no work-related motives, < 50% of motives work-related, ≥ 50% of motives work-related).

**Results:**

Use for relaxation (59.3%), enjoyment (47.2%), social reasons (35.3%), coping (35.1%), medical reasons (30.9%), and sleep (29.9%) were the most common motives. Almost 40% of respondents reported one or more of their cannabis use motives were work-related, with coping (19.9%) and relaxation (16.3%) most commonly reported as work-related. Younger age, poorer general health, greater job stress, having a supervisory role, and hazardous work were associated with increased odds of reporting at least some cannabis use motives to be work-related, while work schedule and greater frequency of alcohol use were associated with reduced odds of motives being primarily work-related.

**Conclusions:**

Cannabis use motives among workers are diverse and frequently associated with work. Greater attention to the role of work in motivating cannabis use is warranted.

**Supplementary Information:**

The online version contains supplementary material available at 10.1186/s12995-024-00424-7.

## Background

Cannabis is widely used around the world. In 2021, an estimated 219 million people used cannabis, accounting for approximately 4% of the global population aged 15 to 64 years [1]. Previous research, conducted primarily among youth, has found enjoyment/fun, conformity, experimentation, social enhancement and celebration, boredom, relaxation, problem avoidance, and perceived relative low risk to be common motives cited for using cannabis [2–6]. As individuals age and transition to adulthood, however, their motives for using cannabis may also change, as the contexts of substance use, social expectations, and responsibilities shift and opportunities for leisure time decrease [7–11]. Consequently, it may not be appropriate to generalize past findings regarding motives for cannabis use among youth to adult populations.

Working adults, in particular, may experience various work-related circumstances that motivate them to use cannabis, such as high work stress, low job satisfaction, and long hours or irregular shifts [12]. Indeed, recent data suggests cannabis use is common in the working population [12–16]. However, little is known about why workers use cannabis. Prior studies of working-aged adults (including a mix of employed and unemployed individuals) have found the primary motives for cannabis use to be relaxation, enjoyment, coping (with stress, anxiety, and depression), enhancing leisure enjoyment, socialization, and increasing creativity and concentration [8, 17–19]. In some studies, workers specifically described using cannabis as a way to detach from work-related concerns, relax at the end of the workday, as a reward for hard work, to cope with work-related stress, to induce sleep, and to enhance their work performance and productivity [7–10, 20]. A more recent study of mental health professionals found specific medical uses as additional motives for using cannabis [21].

However, this previous body of research has been primarily qualitative, based on small samples, and most did not have a primary focus on working adults. Further, it is not clear from previous research the extent to which workers consider their motives for cannabis use to be related to work, nor the characteristics of workers and the workplace that may drive work-related cannabis use motives. With cannabis use among working-aged adults increasing [22] and the legal status of cannabis use continuing to evolve worldwide, there is a need to address these gaps in knowledge regarding cannabis use motives in a working population. Therefore, the objectives of this study are to: (1) determine workers’ motives for using cannabis and whether their motives are associated with work; and (2) explore the personal and work factors associated with having work-related motives for using cannabis.

## Methods

### Study sample

Data for this analysis come from an ongoing research program focused on cannabis use and workplace cannabis use perceptions among Canadian workers [13, 23, 24]. The study sample was recruited in June 2018, approximately four months before the legalization of non-medical cannabis use in Canada. Most of the sample was randomly selected and recruited from two pre-existing panels of Canadians who had previously agreed to participate in periodic surveys. Additionally, a small proportion of the survey sample was randomly selected from the general Canadian population using a traditional random digit dialing frame and approach.

Respondents were eligible to participate if they were 18 years of age or older, currently employed, and working 15 or more hours per week for another person or business employing five or more persons. A total of 2,014 eligible workers responded to the invitation to participate and agreed to participate (1,936 panel respondents, 78 random digit dialing respondents). Of this group, 592 workers reported using cannabis in the past year. Due to small numbers, three workers with non-binary genders or who did not disclose their preferred gender were excluded from the analysis, leaving an analytic sample of 589.

All respondents provided informed consent to participate. This study was approved by the University of Toronto Health Sciences Research Ethics Board (reference number 36019).

### Data collection and measures

Participating workers were administered a survey online (*n* = 479) or by telephone (*n* = 110), depending on respondent preference. The survey collected information on participants’ sociodemographic characteristics, work and workplace characteristics, and cannabis use patterns.

#### Dependent variables

Using questions adapted from the Canadian Tobacco, Alcohol and Drugs Survey [25] and the Canadian Cannabis Survey [26], respondents were asked about the frequency of cannabis use in the 12 months before the survey (ranging from never to five or more days per week). Respondents who indicated using cannabis were asked about their specific motives(s) for use by selecting one or more motives from a provided list (see Additional file 1: Cannabis use motives survey item), developed and informed by prior research on motives for cannabis use [2, 3, 27, 28]. For each motive reported, respondents were then asked a follow-up question to query whether the specified motive was work-related, that is, related to their work or to help them manage in the workplace (yes/no).

We used this information in two ways. First, we categorized the motives for cannabis use into 10 groups based on previous research [2, 3, 28] and study team knowledge. These groupings were enjoyment, conformity, expansion, coping, social, boredom, sleep, relaxation, medical use, and miscellaneous (see Table 1 for the specific motives included in each group). Second, we classified respondents into one of three groups based on their motives for cannabis use: (1) no work-related cannabis motives, where respondents reported all of their motives for using cannabis were unrelated to work; (2) less than 50% work-related motives, where less than half of their motives were reported to be work-related; and (3) at least 50% work-related motives, where at least half of their motives were considered work-related.

Additional data were collected on self-reported purpose of cannabis use (non-medical, medical, mixed) and workplace use, defined as using cannabis within 2 h before work, during work (excluding breaks), during breaks, and/or at the end of a workday at the workplace.

#### Independent variables

*Personal characteristics.* Data on sociodemographic characteristics included age, sex, birth country, and highest level of education achieved. Health-related characteristics, assessed with items from the Canadian Community Health Survey [29], included self-perceived general health (ranging from poor to excellent), current frequency of smoking cigarettes (not at all, occasionally, daily), and frequency of alcohol consumption in the past year (ranging from never/<1 day per month to 4 or more days per week).

*Work characteristics.* Using items from the Canadian Labour Force Survey [30], data were collected on the following work characteristics: job permanence, average number of paid hours worked per week, job tenure, usual work schedule (regular shift [regular daytime/evening/night shift]; non-regular shift [e.g., rotating or split shift, on-call, other irregular schedule]), and workplace size. Additionally, respondents were asked to describe the stress they experienced most days at work in the past 12 months (with response options being not at all stressful, not very stressful, a bit stressful, quite a bit stressful, and extremely stressful) using an item from the Canadian Community Health Survey, an annual cross-sectional survey conducted by Statistics Canada examining the health status, health care utilization, and health determinants of the Canadian population [29].

A new item was developed to identify workers’ participation in hazardous or safety-sensitive work tasks at least weekly in the past 12 months (yes/no). Example tasks were provided, informed by the OHS Vulnerability Measure [31], including, but not limited to: driving a motor vehicle; operating or working near equipment, machinery, or tools; sharps work (e.g., needles, scalpels, scissors, knives); working from heights 2 m/6.5 feet or more above ground; and electrical work that may be a source of electrical shock. An item assessing whether the worker had a supervisory role was also developed for this study. Finally, the degree of supervisor contact in the past year was assessed using an item from Frone and Trinidad (*I have a lot of contact with my supervisor during a typical workday*) [32], with response items ranging from strongly agree to strongly disagree.

### Analysis

A descriptive analysis was undertaken to describe the distribution of cannabis use motives that were reported, the specific motives for cannabis use reported, and whether the motives were work-related. Differences in cannabis use characteristics (frequency of use, purpose of use, workplace use) and personal and work characteristics were examined across the three categories of work-related motives (no work-related motives, less than 50% work-related motives, at least 50% work-related motives) using chi-square tests and ANOVA analyses.

Multinomial logistic regression analyses were conducted to estimate associations between respondents’ personal and work characteristics with work-related cannabis use motives (less than 50% work-related motives, at least 50% work-related motives), compared to no work-related motives, simultaneously adjusting for the effect of all other independent variables. Frequency of cigarette smoking and alcohol consumption, work hours, job tenure, job stress, supervisor contact during the workday, and workplace size were treated as continuous variables. All other variables were treated as nominal. Survey mode (online, telephone) was also included in the model. To address potential overfitting of the regression model, analyses were repeated, excluding covariates not found to be associated in initial multivariable analyses (work hours, job tenure, supervisor contact, workplace size). Results from these models were similar. Final results for the smaller, parsimonious model are presented, with results from the larger model available upon request. All data analyses were undertaken using SAS software Version 9.4 (SAS Institute Inc., Cary, NC, USA).

Among the 589 respondents, 56 were missing data on the outcome (*n* = 33) and/or one or more independent variables (*n* = 23). Multiple imputation was used to address missing data for the regression analyses using a fully conditional specification approach [33] with IVEware version 0.3 [34]. The imputation models contained all variables included in the analytic models and were run using 20 imputation cycles. Model parameters were estimated separately in each imputed dataset and combined with PROC MIANALYZE in SAS. In a sensitivity analysis, model results are reported from complete case analyses (*n* = 533).

## Results

### Motives for cannabis use

The number of motives for cannabis use reported by respondents ranged from 1 to 14 (median 3 motives per respondent). Table 1 presents the motives for cannabis use reported by the sample of respondents. The most common motives reported for using cannabis in the past year included use for relaxation (59.3%), enjoyment (47.2%), and social reasons (35.3%). Use for coping (35.1%; namely for stress and anxiety), medical reasons (30.9%; namely for pain relief), and for sleep (29.9%) were also frequently reported. The motives most often identified as being work-related were coping (19.9%) and relaxation (16.3%). A smaller proportion of respondents also reported that medical (9.7%), enjoyment (8.3%), and sleep (7.1%) motives were work-related.

**Table 1:** Motives for cannabis use reported by workers (*n* = 589)

Motives for cannabis use	Total (*n* = 589)	Motive self-reported as work-related (*n* = 589)^a^
	*n* (column %)^b^	*n* (column %)^b^
**Enjoyment**	**278 (47.2)**	**49 (8.3)**
Feel good / improve my mood	264 (44.8)	48 (8.2)
Enjoy it / have fun	29 (4.9)	--
**Conformity**	**16 (2.7)**	**--**
Felt like I needed to / felt pressured in order to fit in	16 (2.7)	--
**Expansion**	**168 (28.5)**	**34 (5.8)**
Wanted to alter my perspective / think differently	108 (18.3)	13 (2.2)
Enhance my creativity	97 (16.5)	20 (3.4)
Help my concentration	49 (8.3)	18 (3.1)
**Coping**	**207 (35.1)**	**117 (19.9)**
Cope with feelings of depression	77 (13.1)	23 (3.9)
Cope with stress	156 (26.5)	87 (14.8)
Manage anxiety	125 (21.2)	54 (9.2)
Forget my problems	50 (8.5)	17 (2.9)
**Social**	**208 (35.3)**	**20 (3.4)**
Socialize / use in social situations	8 (1.4)	--
Special occasion	178 (30.2)	7 (1.2)
Feel more comfortable in an unfamiliar situation	39 (6.6)	7 (1.2)
Make me feel more confident	25 (4.2)	9 (1.5)
**Boredom**	**82 (13.9)**	**--**
Had nothing better to do / relieve boredom	82 (13.9)	--
**Sleep**	**176 (29.9)**	**42 (7.1)**
Help me sleep / for insomnia	176 (29.9)	42 (7.1)
**Relaxation**	**349 (59.3)**	**96 (16.3)**
To relax	349 (59.3)	96 (16.3)
**Medical use**	**182 (30.9)**	**57 (9.7)**
Relieve physical pain	175 (29.7)	54 (9.2)
Manage other medical condition(s) or symptom(s)	28 (4.8)	--
**Miscellaneous** ^c^	**25 (4.2)**	**--**

^−−^Cell values suppressed where cell size ≤ 5 to protect privacy of respondents

^a^ A total of 33 respondents reported using cannabis in the past year, but were missing data on whether their motives for use were related to work

^b^ Respondents could select multiple motives for their cannabis use. Therefore, column percentages do not add up to 100%

^c^ Includes the following motives: Have to use it / cannot function without using it; To try it, curious to try; Just a habit; Mistake; To replace alcohol

### Features of cannabis use among workers with and without work-related motives

Almost two thirds of respondents (60.1%) reported no work-related motives, while 16.4% reported less than half of their motives to be work-related and 23.6% reported at least half of their motives were work-related. Table 2 compares the features of past-year cannabis use across respondents with and without work-related cannabis use motives. Overall, the frequency of cannabis use was higher among respondents reporting work-related motives compared to those with no work-related motives (*P* < 0.0001). Daily or almost daily use (five or more days per week) was reported by 42.9% of respondents with less than 50% work-related motives and 38.2% of respondents with at least 50% work-related motives, compared to 14.1% of respondents with no work-related motives. Self-reported purpose of use was also significantly different, with a greater proportion of respondents reporting work-related motives also reporting medical only or mixed purpose cannabis use (*P* < 0.0001). Finally, workplace use was more commonly reported by respondents reporting work-related motives (41.8% with less than 50% work-related motives; 49.6% with at least 50% work-related motives) compared to respondents with no work-related motives (12.7%) (*P* < 0.0001).

**Table 2:** Features of cannabis use according to pattern of cannabis use motives (*n* = 589)^a^

Features of cannabis use	Cannabis use motives			*p*-value^b^
	No work-related motives **(** *n* ** = 334)**	**Less than 50% work-related motives (** *n* ** = 91)**	**At least 50% work-related motives (** *n* ** = 131)**	
	*n* (column %)	*n* (column %)	*n* (column %)	
Frequency of past-year use				< 0.0001
< 1 day/month	168 (50.3)	8 (8.8)	27 (20.6)	
1–3 days/month	68 (20.4)	14 (15.4)	23 (17.6)	
1–4 days/week	51 (15.3)	30 (33.0)	31 (23.7)	
5 + days/week	47 (14.1)	39 (42.9)	50 (38.2)	
Self-reported purpose of use^c^				
Non-medical only	260 (79.3)	49 (53.8)	62 (48.1)	< 0.0001
Medical only or mixed	68 (20.7)	42 (46.2)	67 (51.9)	
Workplace use in past year^d^				
Yes	42 (12.7)	38 (41.8)	65 (49.6)	< 0.0001
No	290 (87.3)	53 (58.2)	66 (50.4)	

^a^ A total of 33 respondents reported using cannabis in the past year, but were missing data on whether their motives for use were related to work

^b^ P-values correspond to the results of chi-square analyses comparing respondents with no work-related cannabis use motives, those with less than 50% work-related motives, and those with at least 50% work-related motives

^c^ A total of 8 respondents were missing data on purpose of use

^d^ A total of 2 respondents were missing data on workplace use

### Study sample characteristics

Additional file 2: Study sample characteristics presents the personal and work-related characteristics of the sample, overall and according to the pattern of cannabis use motives in the past year. The average age of the sample was 40.2 years and included more men than women (61.8%). Most of the sample had a permanent job (87.3%), worked a regular shift (82.3%), and worked, on average, 38.4 h per week.

Compared to respondents reporting no work-related cannabis use motives, workers reporting work-related motives were more likely to be younger (on average), in poorer health, and smokers. When looking at their work characteristics, workers reporting at least 50% of their motives to be work-related worked longer hours and were more likely to work in hazardous positions and have a supervisory role. Both groups of respondents reporting work-related motives also were more likely to report greater job stress.

### Personal and work correlates of work-related cannabis use motives

Table 3 presents the findings of a fully-adjusted multinomial logistic regression analysis assessing the associations of personal and work characteristics with work-related cannabis use motives in the past year. Compared to respondents 50 years and older, the odds of reporting work-related motives were higher for respondents 18 to 30 years old (less than 50% work-related: odds ratio [OR] 2.67, 95% confidence interval [CI] 1.47–4.84; at least 50% work-related: OR 3.15, 95%CI 1.56–6.35). The odds of reporting less than 50% motives as work-related were also significantly higher for 31- to 49-year-olds (OR 2.05, 95%CI 1.20–3.48) and non-significantly higher for respondents reporting at least half of their motives as work-related (OR 1.44, 95%CI 0.77–2.71). Respondents born in countries other than Canada were also non-significantly more likely to report work-related motives (less than 50% work-related: OR 1.56, 95%CI 0.84–2.90; at least 50% work-related: OR 1.87, 95%CI 0.90–3.90).

Having poorer health was found to be associated with work-related motives, although non-significantly for the group of respondents reporting at least 50% work-related motives (less than 50% work-related: OR 1.58, 95%CI 1.02–2.46; at least 50% work-related: OR 1.47, 95%CI 0.88–2.46). Finally, among the remaining personal characteristics, more frequent alcohol consumption was associated with reduced odds of reporting at least 50% motives as work-related (OR 0.77, 95%CI 0.60-1.00), with no relationship seen for less than 50% work-related motives.

Among the work characteristics, having a non-regular work schedule was associated with reduced odds of having at least 50% of motives being work-related (OR 0.40, 95%CI 0.19–0.85), while being in a supervisory role (OR 2.14, 95%CI 1.28–3.60) and performing hazardous work (OR 1.63, 95%CI 0.97–2.75) were both associated with greater odds of having at least 50% of motives being work-related. The odds of having work-related motives were non-significantly elevated among respondents with permanent jobs (less than 50% work-related: OR 1.80, 95%CI 0.91–3.56; at least 50% work-related: OR 1.98, 95%CI 0.76–5.14) and significantly higher with increasing job stress (less than 50% work-related: OR 1.30, 95%CI 1.03–1.64; at least 50% work-related: OR 1.49, 95%CI 1.13–1.98). No other personal or work characteristics were associated with work-related motives.

### Results from sensitivity analyses

Results from the multinomial logistic regression analysis using data from respondents with complete data (*n* = 533) were similar in pattern and in the overall interpretation of findings, with minor exceptions relating to statistical significance (see Additional File 3: Results of complete case analysis). In the complete case analysis, poor general health was statistically significantly associated with having less than 50% work-related motives (OR 1.84, 95%CI 1.08, 3.13) and at least 50% work-related motives (OR 1.63, 95%CI 1.02, 2.60), while more frequent alcohol consumption was no longer statistically associated with reduced odds of reporting at least 50% motives as work-related (OR 0.82, 95%CI 0.64–1.04). Performing hazardous work at least weekly was statistically significantly associated with greater odds of having at least 50% work-related motives (OR 1.70, 95%CI 1.06, 2.73), but job stress was no longer statistically significantly associated with the odds of having less than 50% work-related motives (OR 1.16, 95%CI 0.89–1.52).

**Table 3:** Multivariable-adjusted ORs (95% CIs) of work-related cannabis use motives by personal and work-relatedcharacteristics^a^

Characteristics	Less than 50% work-related cannabis use motives	At least 50% work-related cannabis use motives
	OR (95% CI)	OR (95% CI)
Age		
18 to 30 years old	2.67 (1.47, 4.84)	3.15 (1.56, 6.35)
31 to 49 years old	2.05 (1.20, 3.48)	1.44 (0.77, 2.71)
50 + years old	1.00	1.00
Sex		
Female	1.00	1.00
Male	1.33 (0.82, 2.14)	1.09 (0.64, 1.85)
Highest education achieved		
High school diploma or below	1.00	1.00
More than high school	1.35 (0.73, 2.48)	0.92 (0.46, 1.84)
Country of birth		
Canada	1.00	1.00
Other	1.56 (0.84, 2.90)	1.87 (0.90, 3.90)
Self-rated general health		
Very good/Excellent	1.00	1.00
Good/Fair/Poor	1.58 (1.02, 2.46)	1.47 (0.88, 2.46)
Current frequency of cigarette smoking (ordinal)	1.19 (0.90, 1.58)	1.33 (0.95, 1.86)
Past-year frequency of alcohol consumption (ordinal)	0.95 (0.76, 1.19)	0.77 (0.60, 1.00)
Usual work schedule		
Regular day, evening or night shift	1.00	1.00
Non-regular shift (rotating, split, on call, irregular)	1.29 (0.74, 2.26)	0.40 (0.19, 0.85)
Has a permanent job		
No	1.00	1.00
Yes	1.80 (0.91, 3.56)	1.98 (0.76, 5.14)
Performed hazardous work tasks weekly		
No	1.00	1.00
Yes	0.95 (0.60, 1.51)	1.63 (0.97, 2.75)
Has a supervisory role		
No	1.00	1.00
Yes	1.00 (0.62, 1.60)	2.14 (1.28, 3.60)
Job stress (ordinal)	1.30 (1.03, 1.64)	1.49 (1.13, 1.98)

^a^Adjusted for all other factors included in the table

Abbreviations: CI, confidence interval; OR, odds ratio

## Discussion

This study aimed to address an important knowledge gap on why workers use cannabis and how their motives are related to their work. In this sample of workers, the motives reported for using cannabis were diverse and certain motives, namely coping and relaxation, were more often reported as being related to work. Several personal and work characteristics were also found to be associated with work-related cannabis use motives.

Consistent with mostly qualitative studies of working-aged adults [8, 17–19, 21], the most common cannabis use motives reported by workers in this sample were relaxation, enjoyment, coping, socialization, and expansion. Our results also provide further evidence that some motives for use among youth (adolescents and young adults) and working-aged adults differ. Namely, conformity, experimentation, and boredom, while frequently reported by youth [2, 4–6], were much less often reported in this working sample. Unlike prior studies, which were mainly conducted more than 10 years ago, sleep and medical motives were also reported by almost a third of respondents, perhaps reflecting the growing public discourse on the therapeutic potential of cannabis [35, 36]. A recent study conducted by our team also found that workers’ compensation claimants often reported using cannabis to cope with stress and for relaxation, but those who reported using cannabis specifically for their injury and illness symptoms more often reported use for pain, sleep, and mental health [14]. While evidence to support the effectiveness of cannabis for physical and mental health continues to evolve [37], healthcare providers should be aware of these common motivations for use among their working patients in order to best counsel them on use.

Our study extends the limited research in this area by demonstrating the connection between work and workers’ motives for using cannabis. Nearly 40% of workers in this sample reported using cannabis for reasons related to work (including almost a quarter who reported at least half of their motives were work-related), with this use primarily related to coping and relaxation, and to a lesser extent, medical reasons and sleep. Our quantitative data support the findings of prior qualitative studies that found some workers report using cannabis to detach from work-related concerns, cope with work stress, and relax at the end of the workday [7–10, 20]. Due to the nature of the measure, we were unable to disentangle whether work-related motives were the result of workers trying to cope with issues at work (as suggested by these previous studies), enabled them to remain at work (e.g., by managing health symptoms or stress that would otherwise impact work performance) or both. Nonetheless, findings suggest work plays a prominent role in workers’ motives for using cannabis.

Respondents with work-related motives also reported more frequent cannabis use compared to respondents with no work-related motives. Coping motives, most prominent among those reporting work-related motives, have been shown to be associated with more frequent cannabis use [38]. Further, almost half of respondents with work-related motives reported workplace cannabis use, compared to 13% of respondents without work-related motives, suggesting workers motivated to use cannabis for reasons related to their work may be transferring that use to the workplace in an effort to support or manage aspects of their working life. Future research should examine the relationship between work-related cannabis use motives and workplace and health outcomes using longitudinal data.

When examining the correlates of work-related cannabis use motives, several personal characteristics were associated with having work-related motives. In particular, we observed younger workers were more likely to report work-related motives for cannabis use. Conceivably, circumstances commonly experienced by younger workers, including job transitions (e.g., starting new jobs, promotions, career establishment) and balancing life events with work (e.g., child rearing) may influence a worker’s decision to use cannabis. Unfortunately, we lack the data necessary to examine these issues in depth. On the other hand, more frequent alcohol consumption was associated with reduced odds of reporting at least 50% of motives as work-related. This finding suggests workers with work-related motives may be using cannabis as their preferred means of managing with work and are less likely to use alcohol to fulfill this function.

Poorer health and job stress were found to be associated with work-related cannabis use motives. These findings coincide with the self-reported motives for use previously described, whereby workers with these characteristics may be using cannabis to address their health or stress caused by and/or affecting work. However, the cross-sectional study design limits interpretation of the direction of these relationships. Unexpectedly, having a permanent job was associated with a greater likelihood of work-related cannabis use motives, albeit non-significantly. One plausible explanation is that some respondents may be ‘locked’ into permanent jobs despite job dissatisfaction for reasons, such as fear of losing benefits or health insurance, a desire to retain seniority, and a lack of alternative job opportunities [39].

Other work characteristics were only associated with having at least 50% work-related motives, including having a supervisory role and performing hazardous work tasks. It is possible that respondents are using cannabis to cope with the work demands inherent in these roles. Workers performing hazardous work tasks may also be working in jobs prone to injury, leading to pain and poor health, for which workers may turn to cannabis to manage symptoms. In contrast, workers with non-regular work schedules were less likely to report significant work-related motives, potentially due to a weaker attachment to work.

Given the novelty of this study and the lack of empirical studies in this area to compare with, the explanations provided for these findings should be considered tentatively until further research can unpack the findings around work characteristics. More nuanced features of a worker’s job or work environment, such as the presence of supportive working relationships, degree of job demands (including physical, psychological, and time demands), or extent to which a worker perceives control over their job, may be important for explaining work-related motives for use, but were not captured in this study. It will be necessary for future studies to examine a broader range of psychosocial work environment factors in order to better understand the role of work in cannabis use motives. Furthermore, data for this study were also collected before the legalization of non-medical cannabis use in Canada. With an increasing number of working-aged adults now using cannabis [16, 22, 23, 40], future research should examine how workers’ motives for use may also be changing over time.

Our findings should be interpreted in light of the following strengths and limitations. Although the response rate was low (13.2%), it is conservative because the eligibility of those sampled but not contacted, is unknown. Despite this, we recruited a large and diverse sample of Canadian workers who were diverse with respect to both sociodemographic and work characteristics and similar in composition to the Canadian labour force. Respondents also exhibit similar characteristics as adults reporting cannabis use in general population studies [13, 41, 42]. As such, we believe our findings are broadly generalizable, namely descriptive findings on the more common reasons for use and the inferences with respect to the correlates of work-related motives, though the precise prevalence estimates may not be [43]. Social desirability bias may also have led to an underestimate of workers’ cannabis consumption and may have influenced their self-reported motives for use. However, surveys were mainly completed online, likely reducing fears of disclosure.

## Conclusions

This study provides novel insight into the motives for cannabis use among employed adults and demonstrate a considerable proportion of workers perceive their motives to be work-related. Findings also suggest there are important personal and work-related characteristics that distinguish workers using cannabis for reasons related to work. Greater recognition of the role of work in motivating use among working-aged adults is warranted, though more research is needed to understand the workplace factors contributing to cannabis use motives and its outcomes.

### Electronic supplementary material

Below is the link to the electronic supplementary material.

Additional file 1: Cannabis use motives survey item.

Additional file 2: Study sample characteristics, overall and according to the pattern of cannabis use motives in the past year.

Additional file 3: Results from complete case analysis.

## Data Availability

The data that support the findings of this study are available from the corresponding author on reasonable request.

## Abbreviations

CI: Confidence interval
OR: Odds ratio
