# Supplementary material for: Cannabis use motives and associations with personal and work characteristics among Canadian workers: a cross-sectional study
Source: J Occup Med Toxicol. 2024 Jun 13;19:25. doi: 10.1186/s12995-024-00424-7 (PMC11177395; doi:10.1186/s12995-024-00424-7)
Supplement: Supplementary file 1 — Additional file 1: Cannabis use motives survey item. [file 12995_2024_424_MOESM1_ESM.pdf]

ADDITIONAL FILE 1: Cannabis use motives survey item

Carnide N, Chrystoja BR, Lee H, Furlan AD, Smith PM. Cannabis use motives and associations with personal and work characteristics among Canadian workers: A cross-sectional study.

MOTIVES FOR CANNABIS USE

More specifically, for what reasons did you use cannabis? Please select all that apply.

To feel good / improve my mood 1
To forget my problems.. 2
To cope with stress 3
Because I had nothing better to do / to relieve boredom.. 4
Because it was a special occasion 5
Because I wanted to alter my perspective / think differently 6
To enhance my creativity 7
To help with my concentration 8
Because it makes me more comfortable in an unfamiliar situation.......................... 9
Because I felt like I needed to/felt pressured to in order to fit in 10
To make me feel more confident... 11
Because I feel I have to use it / I cannot function without using it 12
To relax... 13
To manage anxiety 14
To cope with feelings of depression 15
To relieve physical pain (e.g., arthritis, back pain, migraine, neuropathic pain)...... 16
To help me sleep / for insomnia 17
To manage a medical condition or medical symptom that is not listed here: please specify
: 18
Other (please specify) : 77
Prefer not to answer 99
