# Supplementary material for: Cannabis use motives and associations with personal and work characteristics among Canadian workers: a cross-sectional study
Source: J Occup Med Toxicol. 2024 Jun 13;19:25. doi: 10.1186/s12995-024-00424-7 (PMC11177395; doi:10.1186/s12995-024-00424-7)
Supplement: Supplementary file 2 — Additional file 2: Study sample characteristics, overall and according to the pattern of cannabis use motives in the past year. [file 12995_2024_424_MOESM2_ESM.pdf]

ADDITIONAL FILE 2: Study sample characteristics

Carnide N, Chrystoja BR, Lee H, Furlan AD, Smith PM. Cannabis use motives and associations with personal and work characteristics among Canadian workers: A cross-sectional study.

Supplementary Table. Personal and work-related characteristics of workers, overall and according to pattern of cannabis use motives (n=589)

		Past-year cannabis use motives[a]			
	All respondents (n=589)	No work-related motives (n=334)	Less than 50% work-related motives (n=91)	At least 50% work-related motives (n=131)	
**Characteristics**	**n (%)**	**n (%)**	**n (%)**	**n (%)**	**p-value[b]**
**Age in years, mean (SD)**	40.2 (12.7)	42.2 (13.3)	38.0 (10.3)	38.6 (12.1)	0.0022
**Age**					0.0067
18 to 30 years old	157 (26.7)	77 (23.1)	22 (24.2)	43 (32.8)	
31 to 49 years old	260 (44.1)	142 (41.5)	52 (57.1)	51 (38.9)	
50+ years old	172 (29.2)	115 (34.4)	17 (18.7)	37 (28.2)	
**Sex**					0.2693
Male	364 (61.8)	199 (59.6)	61 (67.0)	82 (62.6)	
Female	224 (38.0)	134 (40.1)	30 (33.0)	49 (37.4)	
Missing	1 (0.2)	1 (0.3)	0 (0.0)	0 (0.0)	
**Country of birth**					0.4395
Canada	518 (88.0)	301 (90.1)	76 (83.5)	115 (87.8)	
Other	65 (11.0)	33 (9.9)	13 (14.3)	15 (11.5)	
Missing	6 (1.0)	0 (0.0)	2 (2.2)	1 (0.8)	
**Highest education achieved**					0.9971
High school or less	98 (16.6)	56 (16.8)	15 (16.5)	22 (16.8)	
More than high school	490 (83.2)	277 (82.9)	76 (83.5)	109 (83.2)	
Missing	1 (0.2)	1 (0.3)	0 (0.0)	0 (0.0)	
**Self-rated general health**					0.0082
Very Good/Excellent	311 (52.8)	195 (58.4)	40 (44.0)	60 (45.8)	
Poor/Fair/Good	277 (47.0)	139 (41.6)	51 (56.0)	71 (54.2)	
Missing	1 (0.2)	0 (0.0)	0 (0.0)	0 (0.0)	
**Current frequency of cigarette smoking**					0.0767
Not at all	409 (69.4)	247 (74.0)	60 (65.9)	80 (61.1)	
Occasionally	77 (13.1)	39 (11.7)	12 (13.2)	22 (16.8)	
Daily	102 (17.3)	48 (14.4)	19 (20.9)	29 (22.1)	
Missing	1 (0.2)	0 (0.0)	0 (0.0)	0 (0.0)	

		Past-year cannabis use motives[a]			
	All respondents (n=589)	No work-related motives (n=334)	Less than 50% work-related motives (n=91)	At least 50% work-related motives (n=131)	
**Characteristics**	**n (%)**	**n (%)**	**n (%)**	**n (%)**	**p-value[b]**
**Past-year frequency of alcohol use**					0.3087
Never/Less than once a month	94 (16.0)	48 (14.4)	15 (16.5)	28 (21.4)	
1 to 3 times per month	167 (28.4)	92 (27.5)	23 (25.3)	42 (32.1)	
1 to 3 times per week	216 (36.7)	126 (37.7)	34 (37.4)	42 (32.1)	
4 to 7 times per week	110 (18.7)	68 (20.4)	19 (20.9)	18 (13.7)	
Missing	2 (0.3)	0 (0.0)	0 (0.0)	1 (0.8)	
**Usual weekly hours worked, mean (SD)**	38.4 (7.4)	37.8 (6.6)	38.6 (7.5)	40.2 (9.1)	0.0020
**Usual work schedule**					0.3475
Regular shift	485 (82.3)	276 (82.6)	70 (76.9)	110 (84.0)	
Non-regular shift	103 (17.5)	57 (17.1)	21 (23.1)	21 (16.0)	
Missing	1 (0.2)	1 (0.3)	0 (0.0)	0 (0.0)	
**Job tenure[c], mean (SD)**	8.2 (8.8)	8.9 (9.6)	7.5 (7.5)	7.6 (7.7)	0.1122
**Has a permanent job**					0.1352
Yes	514 (87.3)	283 (84.7)	81 (89.0)	119 (90.8)	
No	71 (12.1)	49 (14.7)	9 (9.9)	11 (8.4)	
Missing	4 (0.7)	2 (0.6)	1 (1.1)	1 (0.8)	
**Performed hazardous work tasks weekly**					0.0049
Yes	257 (43.6)	133 (39.8)	35 (38.5)	73 (55.7)	
No	331 (56.2)	200 (59.9)	56 (61.5)	58 (44.3)	
Missing	1 (0.2)	1 (0.3)	0 (0.0)	0 (0.0)	
**Has a supervisory role**					0.0089
Yes	225 (38.2)	116 (34.7)	34 (37.4)	65 (49.6)	
No	359 (61.0)	216 (64.7)	57 (62.6)	64 (48.9)	
Missing	5 (0.9)	2 (0.6)	0 (0.0)	2 (1.5)	
**Frequent contact with supervisor during workday**					0.2134
Strongly disagree	59 (10.0)	32 (9.6)	14 (15.4)	10 (7.6)	
Disagree	98 (16.6)	57 (17.1)	16 (17.6)	22 (16.8)	
Neither agree nor disagree	65 (11.0)	35 (10.5)	8 (8.8)	15 (11.5)	
Agree	175 (29.7)	112 (33.5)	26 (28.6)	31 (23.7)	
Strongly agree	188 (31.9)	96 (28.7)	26 (28.6)	52 (39.7)	
Missing	4 (0.7)	2 (0.6)	1 (1.1)	1 (0.8)	

		Past-year cannabis use motives[a]			
	All respondents (n=589)	No work-related motives (n=334)	Less than 50% work-related motives (n=91)	At least 50% work-related motives (n=131)	
**Characteristics**	**n (%)**	**n (%)**	**n (%)**	**n (%)**	**p-value[b]**
**Job stress**					0.0004
Not at all	26 (4.4)	16 (4.8)	4 (4.4)	6 (4.6)	
Not very stressful	116 (19.7)	78 (23.4)	16 (17.6)	18 (13.7)	
A bit stressful	250 (42.4)	150 (44.9)	39 (42.9)	40 (30.5)	
Quite stressful	143 (24.3)	67 (20.1)	24 (26.4)	44 (33.6)	
Extremely stressful	53 (9.0)	23 (6.9)	7 (7.7)	23 (17.6)	
Missing	1 (0.2)	0 (0.0)	1 (1.1)	0 (0.0)	
**Workplace size**					0.1373
5-19	108 (18.3)	69 (20.7)	17 (18.7)	17 (13.0)	
20 to 99	163 (27.7)	93 (27.8)	24 (26.4)	39 (30.0)	
100 to 499	168 (28.5)	87 (26.1)	21 (23.1)	47 (35.9)	
≥500	150 (25.5)	85 (25.5)	29 (31.9)	28 (21.4)	

[a] A total of 33 respondents reported using cannabis in the past year, but were missing data on whether their motives for use were related to work.

[b] P-values correspond to the results of chi-square and ANOVA analyses comparing comparing respondents with no work-related cannabis use motives, those with less than 50% work-related motives, and those with at least 50% work-related motives.

[c] Data on job tenure missing for 7 respondents (n=5 for respondents with work-related motives, n=1 for respondents with no work-related motives, and n=1 where data on work-related use was also missing).

Abbreviations: CI, confidence interval; SD, standard deviation.
