# Supplementary material for: Cannabis use motives and associations with personal and work characteristics among Canadian workers: a cross-sectional study
Source: J Occup Med Toxicol. 2024 Jun 13;19:25. doi: 10.1186/s12995-024-00424-7 (PMC11177395; doi:10.1186/s12995-024-00424-7)
Supplement: Supplementary file 3 — Additional file 3: Results from complete case analysis. [file 12995_2024_424_MOESM3_ESM.pdf]

ADDITIONAL FILE 3: Results from complete case analysis

Carnide N, Chrystoja BR, Lee H, Furlan AD, Smith PM. Cannabis use motives and associations with personal and work characteristics among Canadian workers: A cross-sectional study.

Supplementary Table. Multivariable-adjusted ORs (95% CIs) of work-related cannabis use motives by personal and work-related characteristics using a complete case analysis (n=533)[a]

Characteristics	Less than 50% work-related cannabis use motives	At least 50% work-related cannabis use motives
	OR (95% CI)	OR (95% CI)
**Age**		
18 to 30 years old	2.64 (1.23, 5.66)	2.85 (1.54, 5.28)
31 to 49 years old	2.77 (1.44, 5.33)	1.09 (0.62, 1.92)
50+ years old	1.00	1.00
**Sex**		
Female	1.00	1.00
Male	1.42 (0.80, 2.51)	1.16 (0.71, 1.90)
**Highest education achieved**		
High school diploma or below	1.00	1.00
More than high school	1.28 (0.61, 2.71)	1.04 (0.55, 1.97)
**Country of birth**		
Canada	1.00	1.00
Other	1.50 (0.71, 3.18)	1.31 (0.64, 2.68)
**Self-rated general health**		
Very Good/Excellent	1.00	1.00
Good/Fair/Poor	1.84 (1.08, 3.13)	1.63 (1.02, 2.60)
**Current frequency of cigarette smoking (ordinal)**	1.22 (0.86, 1.71)	1.26 (0.93, 1.71)
**Past-year frequency of alcohol consumption (ordinal)**	1.01 (0.77, 1.32)	0.82 (0.64, 1.04)
**Usual work schedule**		
Regular day, evening or night shift	1.00	1.00
Non-regular shift (rotating, split, on call, irregular)	1.46 (0.74, 2.90)	0.49 (0.25, 0.96)
**Has a permanent job**		
No	1.00	1.00
Yes	1.71 (0.75, 3.92)	1.73 (0.76, 3.93)
**Performed hazardous work tasks weekly**		
No	1.00	1.00
Yes	0.72 (0.40, 1.28)	1.70 (1.06, 2.73)

Characteristics	Less than 50% work-related cannabis use motives<br>OR (95% CI)	At least 50% work-related cannabis use motives<br>OR (95% CI)
**Has a supervisory role**		
No	1.00	1.00
Yes	1.09 (0.62, 1.94)	1.70 (1.06, 2.72)
**Job stress (ordinal)**	1.16 (0.89, 1.52)	1.57 (1.20, 2.04)

[a]Adjusted for all other factors included in the table.

Abbreviations: CI, confidence interval; OR, odds ratio.
